# Knowledge of Saudi Patients with Autoimmune Diseases about Hydroxychloroquine Toxicity: The Role of Physician–Patient Communication

**DOI:** 10.3390/pharmacy10060152

**Published:** 2022-11-17

**Authors:** Amal Aldarwesh, Ali Almustanyir, Mazoon Alharthi, Duja Alhayan

**Affiliations:** Department of Optometry and Vision Sciences, College of Applied Medical Sciences, King Saud University, Riyadh 145111, Saudi Arabia

**Keywords:** autoimmune diseases, hydroxychloroquine, rheumatoid arthritis, systemic lupus erythematosus

## Abstract

This cross-sectional internet-based questionnaire aimed to assess the knowledge and experience of autoimmune disease patients in Saudi Arabia of the ocular effects of hydroxychloroquine (HCQ). Among the 245 respondents, discontinuation of the drug was linked to its ocular toxicity in approximately 7.3%. Most patients had taken HCQ for a period longer than five years, exceeding a dose of 5 mg/Kg. A lack of education and physician communication about medication toxicity was reported by approximately 40.8% of the participants. Despite the knowledge about HCQ retinopathy, the drug is prescribed to autoimmune disease patients at an inappropriate dosage. Knowledge obtained from physicians’ communication may improve the health outcomes of chronically ill patients. Rheumatologists and ophthalmologists should work together to recognize patients at risk of hydroxychloroquine toxicity and ensure they receive proper education and adhere to periodic follow-up.

## 1. Introduction

Autoimmune rheumatic diseases (ARDs) are progressive inflammatory conditions characterized by joint involvement with a comprehensive profile of systemic manifestations. Currently, there are more than 100 autoimmune diseases that result in altered function of the injured tissue or organ, inducing different types of disabilities, and increasing mortality rates [1]. These conditions include but are not limited to rheumatoid arthritis (RA), systemic lupus erythematosus (SLE), and Sjögren’s syndrome (SS) [2].

Anti-inflammatory drugs and immunosuppressants are the mainstay treatment modalities for affected patients. Currently, hydroxychloroquine (HCQ), is a hydroxylated chloroquine derivative which was designed to treat malaria. HCQ is also used to manage autoimmune conditions including SLE, as monotherapy or in combination with other agents when there are no contraindications [3].

The ocular toxicity of HCQ is known to be directly dose- and time-dependent [4]. The reported ocular side effects include but not limited to loss of visual acuity, photophobia, extraocular muscle palsy, cataract, anterior uveitis, and optic neuritis [5]. However, retinopathy is the most common and serious consequence of HCQ therapy. As HCQ accumulates in the retina, it binds to retina pigment epithelium cells (RPEs), undermining its function as a free radical scavenger and interfering with the autophagy of the photoreceptor outer segment. In a later stage, degeneration of photoreceptors and circular defects around the macula occurs and presents as bull’s eye retinopathy [6,7]. Retinopathy can be prevented through drug toxicity monitoring, which is essential for the safety and prevention of irreversible ocular damage in patients suffering from ARDs. Patient education about the possible progression of their condition, whether due to the disease or medication, could prevent morbidity through improving compliance with therapy and self-care. This is better achieved through physician–patient engagement to promote health literacy. Improving patients’ education and awareness is crucial in chronic, incurable conditions such as ADRs. However, little is known about the education given to the patients about HCQ-induced ocular complications and their level of awareness. We previously reported that ARD patients have a good level of self-efficacy regarding the ocular manifestations of the disease or the side effect of the HCQ [8] This study describes the knowledge and awareness of ARD patients and physician–patient communication about the effect of HCQ on the eye.

## 2. Materials and Methods

### 2.1. Study Population

This was a cross-sectional study using an internet-based questionnaire for patients treated with HCQ. Patients with SLE, RA, or other autoimmune diseases (*n* = 245) who were previously or currently taking HCQ or chloroquine for a period ≤1 year and >1 year between January and June 2021 were invited to complete an online survey. This period was chosen as patients experience side effects such as gastrointestinal upset and headache at the start of the therapy. Tolerance to those side effects develops over time. On the other hand, cutaneous involvement may be reported a few months post-treatment, while retinal toxicity is not commonly experienced before the first five years except if there are predisposing factors. In addition, fear of medication is more likely to be expected for newly diagnosed patients at the initiation of the therapy, which may lead to non-compliance and early discontinuation. Physician–patient communication is essential, especially at the time of diagnosis. Participants were recruited in collaboration with the Saudi Society of Rheumatology and the Charitable Association for Rheumatic Diseases in Riyadh, Saudi Arabia. Patients younger than 18 years of age were excluded from the study.

### 2.2. Ethical Consideration

The study was approved by the ethical committee of the College of Medicine at King Khalid University Hospital, King Saud University. Informed consent was not obtained in written format. Instead, the purpose of the study, the age restriction of participants to 18 and above, and voluntary participation and confidentiality were explained in Arabic at the beginning of the online survey. Answering the survey was considered consent to participation and usage of data.

### 2.3. Sample Size and Data Collection

A free web source calculator (OpenEpi, version 3) was used to determine the sample size Based on the worldwide prevalence, autoimmune diseases affect approximately 8% of the population. Therefore, for a population size of 1,000,000 (one million or more), the hypothesized percentage of the outcome factor in the population (autoimmune disease) was 8%, and at confidence interval (CI) levels of 95% and 97%, the calculated sample size was 114 and 139, respectively. The current survey was answered by 430 participants; among those 245 completed the survey.

The questionnaire was a modified version of a previously published study by Cabral et al. (2020) [9]. It was translated using the standard method (forward/backward translation by two independent translators) to ensure the accuracy of the translation. A pilot study was conducted on patients with ARD to test the functionality of the research tool and identify any language difficulty, especially for the translated English medical terms related to ocular diseases or side effects of the drug. Demographic data were obtained, followed by the primary indication for using HCQ, and the presence of systemic conditions such as hypertension (HTN), diabetes mellitus (DM), and ocular diseases, including diabetic retinopathy, cataract, and macular degeneration, were collected. In addition, the duration of HCQ use, daily dosage (mg/kg/day), and predicted cumulative doses (gram) were calculated using the data gathered. Participants were asked to provide information on patient–physician interactions and knowledge about drug-induced side effects (Appendix A).

### 2.4. Statistical Analysis

Descriptive statistics were calculated. Continuous variables were presented as mean ± standard deviation (SD), whereas categorical variables were presented as absolute and relative frequencies.

## 3. Results

### 3.1. Demographic Characteristics

Demographic data of the patients who participated in the survey are shown in Table 1. Among the 245 participants, 62% were diagnosed with SLE and 28% with RA, while the rest used HCQ for the management of different ARDs. The majority of the respondents were female of childbearing age, with ages ranging from 18 to 75 years old (Table 1).

### 3.2. Hydroxychloroquine Use and Risk of Retinal Toxicity

The majority of patients who participated in this study used HCQ (84.5%), except for two participants (0.8%) who used chloroquine for SLE and another for a non-autoimmune disease, for the management of sickle cell disease. Those who chose “I am not using any of these medications” were directed to the end of the survey. Among those who reported the previous use of HCQ (13.5%), 7.3% stopped taking the medication due to systemic or ocular adverse effects, 4.1% were changed to a different medication class by the treating physician, which is more common in rheumatoid patients, and 2.0% reported that they stopped taking the drug out of fear, without any side effects. Previous users completed the survey and answered questions similar to those who were currently using HCQ to reflect their knowledge during their period of use. There were newly diagnosed patients who had taken the medication for two months up to one year, and patients who had been on the drug for three decades. The average time of HCQ use among all groups is shown in Table 1.

**Figure 1:**
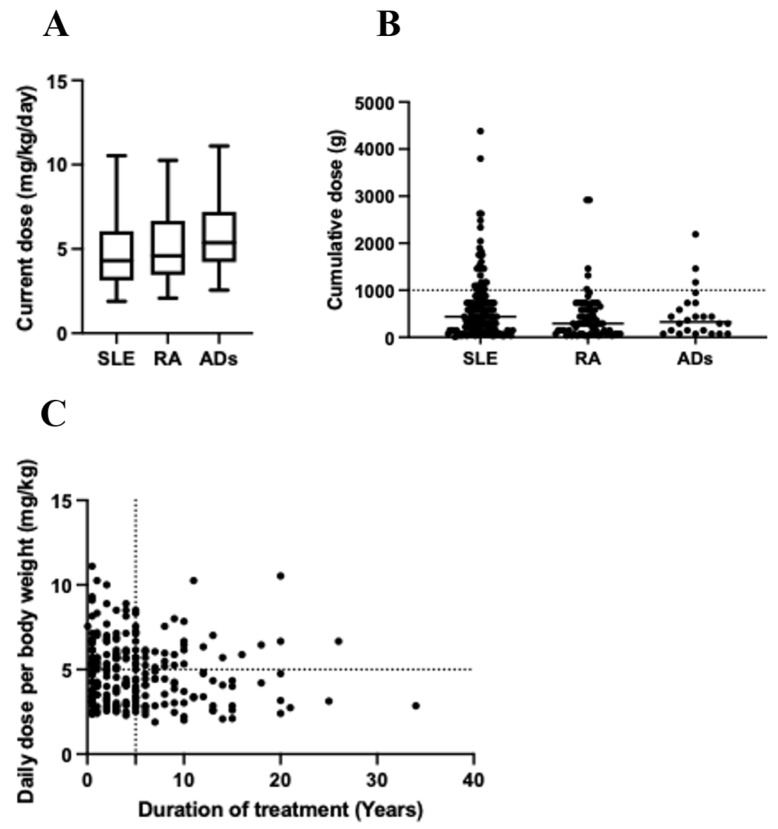
(**A**) Current dose (mg) per day received by participants in the SLE, RA, and ADs groups based on the reported body weight (kg). (**B**) Predicted cumulative dose (g) in the three groups. (**C**) Risk of retinal toxicity for patients exceeding the period of 5 years and daily dose of 5 mg/kg.

The mean HCQ dose for study participants was 4.89 ± 1.9 mg/kg. Several patients (n = 5) in the study reached a critical dose of 10 mg/kg, indicating a risk of toxicity. A higher proportion of patients (48.9%) exceeded 5 mg/kg, as shown in Table 1 and Figure 1A, which is associated with an approximately 10% risk of retinal toxicity within 10 years and an almost 40% risk after 20 years. The current dose (mg) per body weight for each group is shown in Figure 1A. Exceeding a cumulative dose of 1000 g is linked to the duration of the treatment and is more likely to be detected after 5 years of HCQ use. This was observed in 16.3% (n = 40) of the 245 participants (Figure 1B). The detection of patients who exceeded 5 years and 5 mg/kg is shown in Figure 1C. These values were based on the current doses reported by the patients, and the fluctuation in HCQ dose during the treatment duration was not considered.

Other risk factors for retinal toxicity include concomitant systemic and ocular conditions. Only two patients reported the presence of kidney disease; one stated that she was suffering from renal failure and hypertension but still took two HCQ tablets, which made her doses reach 10.3 mg/kg/day. Other chronic conditions included systemic HTN and DM, and a few reported ocular diseases such as glaucoma, cataracts, and macular degeneration. The patient who reported glaucoma as a chronic condition received HCQ at a dose of 6.5 mg/kg/day.

When patients were questioned if they were told about any changes in the retina during a routine examination by an ophthalmologist or optometrist, 20 (8.2%) answered “Yes”; 109 (44.5%) answered “No”; and 16 (6.5%) said “I do not know”. Those who answered “Yes” had an average use of HCQ of 11.3 ± 9.3 years and an average accumulative dose of 1011.6 ± 917.4 g. Incidentally, 11 patients (4.5%) reported a color deficiency discovered during adulthood. Two of those patients said “Yes” about being informed of retinal changes during a clinic visit. Among the 11 patients, the selected nine who described retinal toxicity in their own words are displayed in Table 2.

### 3.3. Hydroxychloroquine Side Effects

The majority of the patients (63.8%) reported more than one adverse symptom. Skin discoloration, pigmentation, and headache were reported (n = 73), followed by blurred vision (n = 65), tinnitus (n = 45), eye pain (n = 42), and eye redness (n = 32). Five patients reported gastrointestinal side effects.

### 3.4. Knowledge and Awareness about HCQ Ocular Toxicity

Patients were asked if their physicians provided information about the autoimmune disease and its possible complications at the time of diagnosis. The majority (69.4%) answered “Yes”; 7.4% answered “Yes, but I forgot it”, and the remaining patients (23.3%) said “No”. A similar question was asked about the ocular effects of HCQ. The majority (53.1%) said “Yes”; and 40.8% answered “No”, while the minority (7.35%) stated that they think they were informed, but they forgot. Similar figures were obtained when patients were asked if their treating physicians referred them to an ophthalmologist at the start of the treatment. More than half of the respondents (59.2%) answered “Yes”, and the rest (40.8%) answered “No". When asked if they were advised by rheumatologists to follow up with an ophthalmologist at least yearly, around half (54.3%) agreed that their rheumatologists advised them to follow up with an ophthalmologist at least yearly, while 33.5% said “No”, and 12.2% heard this information from other sources; these sources included supportive groups on social media (Twitter, Instagram, and WhatsApp), searching the internet, and reading drug pamphlets. One patient knew when she suffered from ocular pain, while one patient’s experience was linked to the onset of diabetes upon visiting an ophthalmologist, and another patient knew she had retinal toxicity after being prescribed a dose above her appropriate weight. One patient even explained that she found out “The day she filled out this survey”. Despite the knowledge about the necessity of ocular examination, very few followed up with an ophthalmologist when they started the medication (7.3%), others never followed up (13.8%), “a recent visit this year” was stated by 26%, “a visit a year ago” was confirmed by 24.9%, and 26.9% answered “a long time ago”.

## 4. Discussion

Autoimmune diseases affect women in all age groups more than men, and they are the leading cause of death in the USA, the UK, and various other countries [10,11]. Specifically, SLE and RA are associated with mortality and morbidity in the affected patients. HCQ has become a long-term standard therapy, especially for patients with SLE. This study presented data for HCQ users, primarily middle-aged women affected mainly by SLE. In Saudi Arabia, the prevalence of SLE was reported as 19.28 per 100,000 [12].

In addition, the study discussed the appropriateness of the drug dosage in relation to the major side effect of retinal toxicity. The prevalence of retinopathy varies but is estimated to be 0.5–2% in long-term users [13,14], compared with a previously reported prevalence of 7.5% among HCQ users in which retinal toxicity was detected before bull’s eye maculopathy was visible [15]. However, there is an agreement that patients with a daily dose of more than 6.5 mg/kg of ideal body weight (IBW)/day and a duration of use of more than five years have a higher tendency to develop retinopathy [13,14,15]. These guidelines were revised by the American Academy of Ophthalmology to recommend a maximum daily HCQ use of ≤5.0 mg/kg actual body weight [16,17]. The present study showed that 48.9% of patients who answered this survey currently exceeded a daily dose of 5.0 mg/kg. Similar findings were reported recently by Al Adel et al. (2021), as approximately 22% of Saudi patients involved in the study were found to be on doses of more than 5 mg/kg, indicating the necessity of reducing HCQ dosages [18]. It is difficult to estimate the duration of inappropriate dosing without retrospective examination of each case; however, this adds to the evidence that treating physicians continue to prescribe high doses of HCQ to their patients who are treatment-dependent for years despite knowing the risks [14,15,18].

Several patients reported a lower body weight of approximately 40 kg, while others had a higher weight of 100 kg, and in both cases, it was observed that patients were receiving an inappropriate dose of HCQ. Our survey reported 23 patients who were told about retinal changes during a visit to an ophthalmologist. Among those patients, nine had retinal involvement that led to discontinuation of HCQ therapy. This yields an increased rate of 3.7% retinal toxicity among the studied samples of long-term HCQ users. Five of these patients exceeded a cumulative dose of 1000 g, as they surpassed a duration of 5 years. Sudden deterioration of vision and development of bull’s eye maculopathy were reported in patients who received a maintenance dose as low as 200 mg for more than ten years [19,20], as well in cases with shorter duration of use [21,22,23]. In this study, one patient with SS reported retinal toxicity within three years of the therapy but received a high dose of HCQ.

The proposed mechanism of maculopathy could be attributed to the accumulation and binding of HCQ to melanin in the REP cells, which adversely affects the metabolism of these cells [6,7] and causes thinning of retinal layers [24,25]. Eventually, this causes central vision loss, reading difficulty, and reduced color perception [26]. Studies have reported that HCQ affects the photoreceptors, which explains the ability of the drug to affect color vision [6,27]. In this study, 11 patients reported difficulty in color discrimination, but they were reluctant to mention more details. Among those patients, a 50-year-old woman with SLE had color discrimination difficulty after approximately 20 years of HCQ treatment, and two patients were informed by the ophthalmologist about the existence of their retinal changes. It has been postulated that undesired side effects are often reversible with drug discontinuation. However, studies have reported that ocular toxicity, due to prolonged use of chloroquine and HCQ, is sustained despite drug cessation [26,28]. Similarly, a case report of Caucasian patients with SLE had bull’s eye maculopathy within four years of starting therapy [29]. Despite the discontinuation of HCQ, retinal damage had progressed three years later. This toxicity could be augmented by chronic debilitating conditions, such as renal failure [17,29]. In fact, two patients in our sample reported concomitant kidney disease, one of whom indicated renal failure.

Although retinopathy is considered a worse outcome of HCQ therapy, some patients may not tolerate other side effects. Skin discoloration was among the most reported complaint of HCQ in our study, which necessitated discontinuation of treatment for some patients. Other reported side effects, which could have been temporary at time of treatment initiation, were gastrointestinal symptoms, blurred vision, ocular pain, headache, and tinnitus.

Patient–doctor interaction and communication improve patients’ awareness about the nature of their disease, thereby improving adherence to medications and self-efficacy [30]. The results showed that 64.4% of patients were educated about their condition, but 40.8% were not informed about the possible effect of HCQ on the eye. The Royal College of Ophthalmologists recommends that patients be given written information on hydroxychloroquine retinopathy and screening for hydroxychloroquine retinopathy in addition to verbal communication [30].

Approximately 33.5% of patients on long-term HCQ treatment were not advised to check their eyes annually, and a smaller proportion received this information from media sources (12.2%). Receiving information from a professional source during visits may convey a better message to patients that reinforces positive behavior in treatment adherence. It has been found that educated patients who were newly diagnosed with RA were more compliant and had lower functional impairment and less pain in follow-up visits [31]. There is no doubt that awareness and knowledge play an essential role in patients’ outcomes in chronic debilitating conditions. However, physicians bear the responsibility for referring patients eligible for screening to the ophthalmology clinic. The current findings have potential limitation because of the study design. The cumulative and daily dose of HCQ was calculated based on the reported body weight and current dosage of the drug regardless of changes in prescribing throughout the duration of the disease, which limits the accuracy of the cumulative dose and the estimation of retinopathy risk.

## 5. Conclusions

Hydroxychloroquine-induced retinopathy is a persistent issue that needs to be recognized, monitored, and reported in patients with autoimmune disease. A high daily intake disproportionate to body weight taken over a long duration increases the risk of ocular toxicity. Rheumatologists and ophthalmologists should work together to recognize patients at risk, maintain them at the appropriate dose, and apply the screening protocol during treatment.

## Figures and Tables

**Table 1:** Demographics and patients’ characteristics (n = 245).

	Condition	SLE	RA	Other ADs
Patient characteristics, No. (%)				
	n	152	68	25
Sex	Male	7 (4.6)	2 (2.9)	1 (4)
	Female	145 (95.4)	66 (97.1)	24 (96)
Education	Primary School	4 (2.6)	1 (1.5)	0
	Secondary School	5 (3.3)	1 (1.5)	2 (8)
	High School	25 (16.4)	17 (25)	4 (16)
	Diploma	14 (9.2)	6 (8.8)	2 (8)
	Bachelor’s degree	84 (55.3)	39 (57.4)	13 (52)
	Higher Education	20 (13.2)	4 (5.9)	4 (16)
Age (years), mean (SD)		32.8 (9.1)	37.4 (10.4)	37 (10.3)
Age at onset of the disease, mean (SD)		25.5 (9.3)	31.2 (10.7)	30.99 (11.6)
Weight (kg), median (IQR)		65 (55.9–75)	66 (56–76)	65 (49.8–75)
Drug Use				
Current daily HCQ dose (mg), median (IQR)		400 (200–400)	400 (200–400)	400 (350–400)
Current daily HCQ dose/weight (mg/kg), mean (SD)		4.8 (1.9)	4.9 (1.8)	5.4 (4.3–6.5)
Duration of the disease (years), mean (SD)		7.3 (6.6)	6.3 (6.3)	5.4 (5.9)
Duration of HCQ use (years), mean (SD)		7.2 (6.4)	4.1 (4.2)	4.04 (4.1)
Predicted cumulative HCQ dose (g), mean (SD)		656.3 (687.4)	451.6 (541.4)	486.7.5 (517.8)
No. at risk (n)				
<4.0 mg/kg		59	24	5
4.0–5.0 mg/kg		28	14	5
>5.0 mg/kg		65	40	15
Exceeding 1000 g cumulative dose		32	5	3

Abbreviations: SLE, systemic lupus erythematosus; RA, rheumatoid arthritis; ADs, autoimmune diseases; HCQ, hydroxychloroquine; IQR, interquartile range (25th–75th).

**Table 2:** Characteristics of patients with definite retinal toxicity.

No.	Sex/Age (years)	Weight (kg)	Systemic/Ocular Disease	Rheumatologic Diseases	Duration of Use (years)	Dose/day (mg/d)	Cumulative Dose (g) *	Daily dose/kg/d (mg/kg/d)	Patient’s Own Statement about Retinal Toxicity
1	F/60	60	Hypertension	SLE	14	400	2044	6.7	I have changes in the right retina because the dose was not suitable for my weight
2	F/39	60	-	SLE	26	400	3796	6.7	I have retinal detachment
3	F/29	72		SLE	20	200	1460	2.8	I have pigmentation in the vision center
4	F/41	59	-	SLE	11	200	803	3.4	I have changes in the retina
5	F/42	66		SLE	3	200	219	3	I have changes in the retina
6	F/51	70	Hypertension, cataract	SLE	34	200	2482	2.9	I see black dots floating in my visual field
7	F/34	77	-	SLE	14	200	1022	2.6	I see black dots floating in my visual field
8	F/38	40	-	RA	9	200	657	8	I have pigmentation in the retina
9	F/47	48		Sjögren’s Syndrome	3	400	438	8.3	Plaquenil caused a little damage to my retina

Abbreviations: AD, autoimmune diseases; SLE, systemic lupus erythematosus; RA, rheumatoid arthritis; SS, Sjögren’s syndrome. * The estimated cumulative dose for the patient assuming that that the dosage is fixed over the duration of the disease.

## Data Availability

Not applicable.

## Appendix A. Questionnaire Used in the Study

**Age (years)**		Gender Male Female	Weight (Kg)	
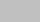
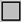
**Educational level**		Elementary High school Diploma Bachelor Higher education		
**1. Which of the following diseases do you have?**		Systemic Lupus Erythematosus (SLE) Rheumatoid Arthritis (RA) Other autoimmune I don’t suffer from an autoimmune disease (***point of exit***)		
**2. When were you diagnosed?**		Please specify		
**3. Do you have chronic conditions?**		Diabetes type 1 Diabetes type 2 hypertension Glaucoma cataract diabetic retinopathy macular degeneration Other please specify		
**4. Have you been taken one of the following medication?**		Chloroquine (Aralen^®^) Hydroxychloroquine (Plaquenil^®^) Other: Yes, I have been taken it No, I have never taken it (***point of exit***) No, however, I have been taken it before. (how long) I quit taking it because: I had complications: Ocular complications please specify Other complications…………………please specify I was afraid of taking it Doctors recommended a new medication…………………please specify		
**5. How long have you been taking the medication?**		Six months or less. 1 year 2 years 3 years 4 years 5 years more…specify		
**6.** **How many pills do you take a day?**		1 2 3 Other dose		
**7. Did your doctor provide you orientation about the disease and its possible complications?**				
Yes, he/she did. I know about it Yes, but I forgot it. No, he/she did not				
**8. Did your doctor provide you orientation about the medication and its effects on the eye?**				
Yes, he/she did. I know about it Yes, but I forgot it. No, he/she did not				
**9. Did your doctor refer you to an ophthalmologist or optometrist for ocular examination at the start of the treatment with hydroxychloroquine?**				
Yes, he/she did No, he/she did not				
**10. Did you know it is necessary to be accompanied by an ophthalmologist or optometrist at least once per year?**				
No, I did not. My doctor did not tell me about that Yes, I did. My doctor explained to me about that. Yes, I heard about it from				
**11. When was the last time you saw an ophthalmologist or optometrist?** When I started the medication This year last year long time ago Before I started the medication I have never seen an ophthalmologist or optometrist				
**12. Do you use any eyeglasses or contact lenses?** Yes. No **13. Did you use the glasses before or after starting treatment with this drug:** Before After **14. What was the diagnosis of your last eye examination?** Myopia Hyperopia Astigmatism other **15. Did the ophthalmologist or optometrist inform you that you have any abnormality in the retina?** Yes, No, I don’t know				
**16. Do you have color vision deficiency?** Yes. No **If Yes, at what age did you become aware (write age)**				
**17. Have you ever had any side effects during taking the medication? Which one?**				
** No, I have not**	Yes, I have: (choose all applicable symptoms) headache. red eyes Color vision discrimination difficulty tinnitus eye pain Others altered skin color blurred vision			
